# Psychosocial therapy for Parkinson's-related dementia: study protocol for the INVEST randomised controlled trial

**DOI:** 10.1136/bmjopen-2017-016801

**Published:** 2017-06-19

**Authors:** Sheree A McCormick, Kathryn R McDonald, Sabina Vatter, Vasiliki Orgeta, Ellen Poliakoff, Sarah Smith, Monty A Silverdale, Bo Fu, Iracema Leroi

**Affiliations:** 1 Division of Neuroscience and Experimental Psychology, University of Manchester, Manchester, UK; 2 Manchester Academic Health Science Centre, Manchester, UK; 3 Greater Manchester Mental Health NHS Foundation Trust, Manchester, UK; 4 Division of Psychiatry, University College London, London, UK; 5 Faculty of Health Studies, University of Bradford, Bradford, UK; 6 Greater Manchester Neurosciences Centre, Salford Royal NHS Foundation Trust, Salford, UK

**Keywords:** feasibility and exploratory study, pilot trial, complex intervention, psychosocial therapy, quality of life, parkinson's disease dementia (PDD), mild cognitive impairment in PD (MCI-PD), dementia with Lewy bodies (DLB), process analysis.

## Abstract

**Introduction:**

Parkinson's disease (PD) with mild cognitive impairment (MCI-PD) or dementia (PDD) and dementia with Lewy bodies (DLB) are characterised by motor and ‘non-motor’ symptoms which impact on quality of life. Treatment options are generally limited to pharmacological approaches. We developed a psychosocial intervention to improve cognition, quality of life and companion burden for people with MCI-PD, PDD or DLB. Here, we describe the protocol for a single-blind randomised controlled trial to assess feasibility, acceptability and tolerability of the intervention and to evaluate treatment implementation. The interaction among the intervention and selected outcome measures and the efficacy of this intervention in improving cognition for people with MCI-PD, PDD or DLB will also be explored.

**Methods and analysis:**

Dyads will be randomised into two treatment arms to receive either ‘treatment as usual’ (TAU) or cognitive stimulation therapy specifically adapted for Parkinson's-related dementias (CST-PD), involving 30 min sessions delivered at home by the study companion three times per week over 10 weeks. A mixed-methods approach will be used to collect data on the operational aspects of the trial and treatment implementation. This will involve diary keeping, telephone follow-ups, dyad checklists and researcher ratings. Analysis will include descriptive statistics summarising recruitment, acceptability and tolerance of the intervention, and treatment implementation. To pilot an outcome measure of efficacy, we will undertake an inferential analysis to test our hypothesis that compared with TAU, CST-PD improves cognition. Qualitative approaches using thematic analysis will also be applied. Our findings will inform a larger definitive trial.

**Ethics and dissemination:**

Ethical opinion was granted (REC reference: 15/YH/0531). Findings will be published in peer-reviewed journals and at conferences. We will prepare reports for dissemination by organisations involved with PD and dementia.

**Trial registration number:**

ISRCTN (ISRCTN11455062).

**Strengths and limitations of this study**

- The use of psychosocial therapies for cognitive impairment in movement disorders is important and under-researched.
- This study uses a range of process and exploratory measures to ascertain the feasibility of undertaking a large efficacy randomised controlled trial powered to assess a range of complex outcomes in both affected participants and their companions.
- The efficacy of the intervention in improving cognition will be evaluated.
- Dyads will be recruited from multiple clinical sites to reduce potential bias when recruiting from a single site.
- Recruiting to target will be a challenge, due to the complexity of the intervention, the relative frailty of some participants and the existing responsibilities of the companion.

## Introduction

### Background and rationale

Within the spectrum of Lewy body disorders, cognitive impairment can manifest as Parkinson's disease (PD) dementia (PDD), Parkinson's disease with mild cognitive impairment (MCI-PD) and dementia with Lewy bodies (DLB). Only very limited drug-based treatments are available for PDD, and no medications have been licensed for MCI-PD and DLB. Without adequate management of the non-motor aspects of these conditions, the risk of being admitted to care is very high. Increasing the availability and the evidence base for non-drug-based therapies for dementia and mild cognitive impairment, such as psychosocial interventions, is a key objective of England's National Dementia Strategy^1^ and other national dementia policy drivers. Unfortunately, there is almost no evidence to support their use in people with more complex forms of dementia, such as dementias associated with Parkinson's-related disorders.^2^ Thus, there is a need to extend psychosocial therapies to this population. Unpublished data provided by public and patient involvement (PPI) representatives and Parkinson's expert consultees involved in this project support this view.

For people with dementia unrelated to Parkinson's-related disorders, psychosocial therapies, such as reality orientation or reminiscence therapy, have been in use for some time.^3^ Recently, in the UK, several large-scale multicentred trials of psychosocial interventions for dementia have been conducted, for example, goal-oriented cognitive rehabilitation^4^ and individual cognitive stimulation therapy (iCST)^5^; however, these have either specifically excluded people with PD/DLB or not been tailored to meet their needs. One of the most widely accepted psychosocial therapies for dementia, which has been accepted for use in the National Health Service (NHS) and is part of the National Institute for Health and Care Excellence^6^ guidelines for dementia care is structured group cognitive stimulation therapy (CST).^7^ Based on these recommendations, as well as its relatively widespread use and availability, CST was chosen by the research team as a candidate therapy to adapt for individuals with Parkinson's-related dementias.

CST was originally developed from a Cochrane systematic review of psychological interventions for people with dementia^8^ and has been shown to be cost-effective, to improve quality of life and to enhance cognition to an extent comparable with trials of cholinesterase inhibitors.^5–7^ CST is based on the principle that stimulating engagement in cognitive and social activity enhances cognitive function and quality of life. However, in spite of the success of the therapy in a group setting, it was recognised that not all people with dementia may have access to, or wish to participate in, group activities. CST was therefore adapted for home-based individualised administration (via a study companion) in the form of ‘individual CST’ (iCST).^5^

The efficacy of iCST was recently investigated in a 26-week multicentre, single-blind, randomised controlled trial (RCT).^5^ The findings revealed a significant improvement in the quality of the relationship of the person with dementia and their study companion, as well as an improvement in health-related quality of life for companions (designated ‘caregivers’). Furthermore, iCST was found to be feasible for people who found travel a barrier to participation. Outcomes related to those who support or care are particularly important, since the perceived burden of care has been shown to be significantly related to the extent of associated cognitive impairment in the person living with Parkinson's-related dementia.^9^ This may be in part due to the added responsibility of managing the impact of concurrent cognitive, psychiatric and physical impairments in the cognitively impaired person. Nonetheless, in spite of the positive findings mentioned above, iCST did not appear to improve cognition or quality of life of the cognitively impaired participants themselves.

While CST as a psychosocial intervention for people with Parkinson's-related dementia has potential, early consultation with PPI and Parkinson's expert groups suggested the need for adaptation. Thus, the first step was to undertake a ‘therapy adaptation’ process in order to meet the specific needs of people with PDD, MCI-PD or DLB. This will be described in detail in a subsequent paper. The adaptation was deemed necessary due to the added complexity of the symptoms related to PD which may include frequent episodes of confusion, marked hallucinations and delusions, high rates of apathy, a myriad of motor symptoms and dependency on companions due to physical disability. It was hypothesised that cognitive stimulation therapy specifically adapted for Parkinson's-related dementias (CST-PD) may improve cognitive function and quality of life in this population and could potentially have a positive effect on companions and other outcomes.

The protocol outlined here describes a feasibility and exploratory study embedded within a pilot trial of CST-PD, which will be delivered by companions. This study will address some of the iterative steps in the process involved in developing a complex intervention for people with dementia, as outlined by the UK's Medical Research Council (MRC) guidance on the topic.^10^ First, it is a test to explore whether it is possible to implement and evaluate this particular complex intervention, including aspects of treatment implementation and dosing, and second, it will be a small-scale trial of the intervention to estimate the treatment effect and its variance on a single main outcome as well as exploratory subsidiary outcomes.

### Overall aim

The purpose of this work is to gather the information needed to design a subsequent full-scale, definitive RCT powered to evaluate the efficacy of CST-PD in improving a range of outcomes relevant to people with PDD, MCI-PD or DLB and their companions.

### Objectives

To fulfil our aim, we will achieve the following objectives:

Primary objectives

- An evaluation of the operational aspects of the study including recruitment, acceptance of the intervention and assessments, and loss to follow-up;
- A process evaluation of the intervention including whether the intervention can be delivered, received and enacted as intended,^11^ as well as acceptability and tolerability of the intervention by the dyads (people with MCI-PD, PDD or DLB, referred to as participants, and their study partners, referred to as companions).

Secondary objectives

- An exploration of the potential efficacy of the intervention on a single primary outcome, cognition;
- An exploration of the potential possible outcome measures in terms of feasibility, clinically meaningful change and relationship to the intervention.

### Research questions and hypotheses

The following are research questions for the feasibility and exploratory study:

- Is it possible to recruit sufficient dyads in a timely manner?
- What is the acceptability and tolerability of the intervention by dyads?
- Which are the most clinically meaningful and acceptable outcomes by which to assess efficacy?
- Based on the findings, what would the sample size of a fully powered definitive RCT of the intervention be?

The following is the hypothesis to explore potential efficacy of the intervention:

- In people with PDD, MCI-PD or DLB, the change in cognitive function from baseline to withdrawal of CST-PD at 12 weeks will favour the intervention compared with treatment as usual (TAU).

## Methods

**Figure 1:**
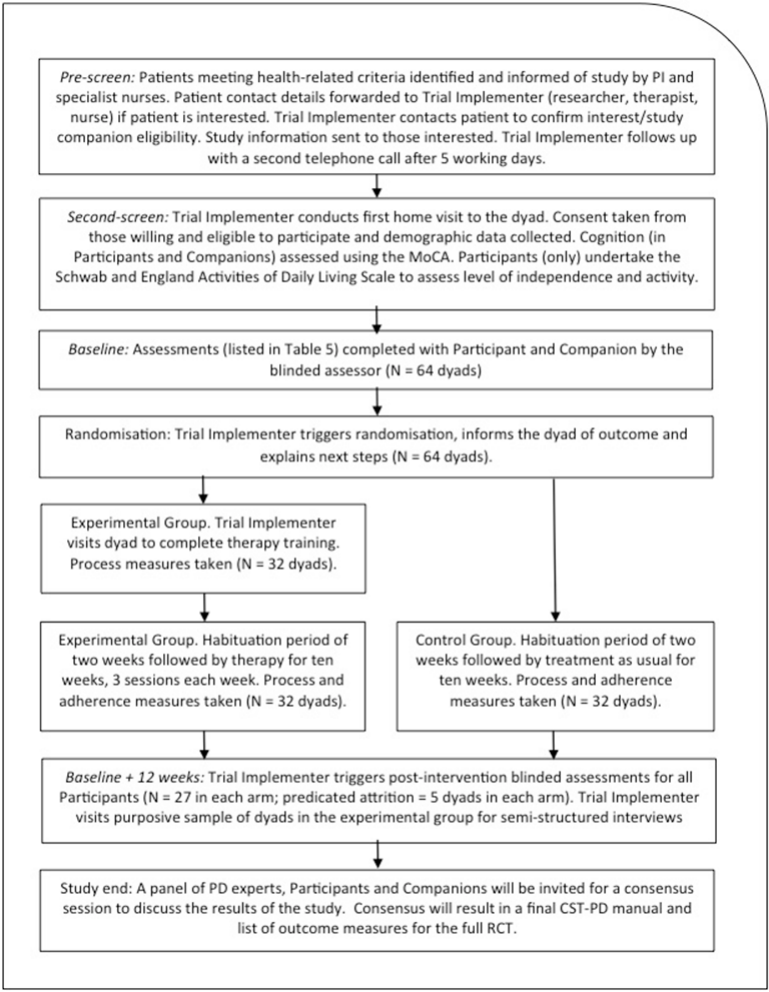
CONSORT-style flow chart.

This protocol was prepared in accordance with SPIRIT 2013 statement^12^ (checklist is available as an online supplementary file). The trial is registered with the ISRCNT (ISRCTN11455062); all study-related items from the WHO Trial Registration Data Set are available at the registry. The protocol lies within a research programme comprising a series of sequential steps in the development, feasibility testing, piloting and eventual full-scale trialling of a complex psychosocial intervention for people with PDD, MCI-PD or DLB. The programme has followed the UK's MRC's updated Guidance on ‘Developing and Evaluating Complex Interventions^10^ and is now at the feasibility, exploratory and pilot trial stage, which we describe here. Specifically, this part of the programme is a fully controlled randomised single-blind exploratory pilot trial with an embedded feasibility process evaluation and evaluation of new outcomes. The protocol for the process evaluation is not reported here but will form the basis of other papers. The pilot trial is designed and powered to test the hypothesis that CST-PD is superior to TAU for people with PDD, MCI-PD or DLB in improving cognitive function when assessed at baseline and 12 weeks post-randomisation. The therapy will be delivered in participants’ homes with recruiting sites in locations in Greater Manchester and other regions in the UK. A CONSORT-style flow chart for the trial is shown in figure 1.

10.1136/bmjopen-2017-016801.supp1 Supplementary material 1

The feasibility study is designed to (1) ascertain the feasibility of conducting the trial in a small scale and (2) explore secondary outcomes for which the trial is not powered to detect efficacy. It is vital to address these issues, since in the case of an intervention such as CST-PD, there are multiple components working in synergy to produce change, and it is not clear at the outset which components may result in change and at which level this may occur (eg, individual, companion, relationship and so on).^10^ Thus, the ‘dimensions of complexity’ highlighted in the MRC's new guidance for developing and evaluating complex interventions^10^ that need to be considered and accounted for in the trial design are outlined in table 1. A single team will evaluate the three sets of outcomes (efficacy trial, feasibility study and exploratory study) in order to foster an ongoing process of integration of evidence as to the conduct of the different steps in the study and trial, as suggested by Moore *et al*.^13^

**Table 1:** Description of ‘dimensions of complexity’ in the adapted study as per MRC's guidelines^10^

Dimension	Reason for complexity
Number of and interactions between components within the experimental interventions	Since the intervention will be delivered by the companion and we anticipate that no two companions or participants will have the same skills, interests or deficits, there may be a degree of variation in each therapy session. The therapy is based on this assumption and is designed to maximise outcomes by adopting a person-centred approach. Participants choose the activities they are most interested in from a library of over 60 cognitively stimulating topics. Companions subsequently personalise the activity through verbal, visual, tactile, auditory, gustatory or olfactory cues. Thus, the therapy subscribes to a structured, yet tailored, approach, delivering an intervention that is standardised with respect to dose and delivery but variable with respect to content.
Number and difficulty of behaviours required by those delivering or receiving the intervention	Standardised companion training is critical to minimise heterogeneity. All companions will be trained to criterion and their skills monitored over time. This element of treatment fidelity will reduce the likelihood of non-significant results at the end of the study being attributed to poor training rather than an ineffective intervention. Companions will receive a minimum of 2 hours training and must have sufficient cognitive function (by not meeting clinical criteria for dementia) to be able to deliver the therapy. Companions’ ability to deliver the therapy will be self-assessed and researcher rated at the end of the training and monitored throughout the study.
Number and variability of outcomes	Although the trial is powered to assess cognitive functioning as the main outcome, it is likely that there may be effects on different outcomes such as behaviour, companion variables, relationship features and health economic issues.
A good theoretical understanding is needed of how the intervention causes change, so that weak links in the causal chain can be identified and strengthened	The background literature of evidence of potential mechanisms, as well as clinical experience, has suggested that each function of the intervention can be linked to identifiable intermediate impacts and final outcomes and can be outlined in a logic model^32 33^ which will be outlined elsewhere.

MRC, Medical Research Council.

### Dyad selection

Sixty-four dyads will be asked to participate. Inclusion and exclusion criteria are listed in table 2.

**Table 2:** Inclusion and exclusion criteria for participants and companions

**Inclusion criteria—participant**	**Exclusion criteria—participant**
Have a diagnosis of possible or probable PDD, MCI-PD or DLB. Diagnosis will be based on standard clinical diagnostic criteria^34–36^ determined by the referring clinician and verified on screening.	Lack sufficient physical and mental capability to participate in the therapy. Capability will be based on clinical impression at the initial screening visit and informed by scores obtained on the Schwab and England Activities of Daily Living Scale^22^ and MoCA.^21^
Be willing to participate in 30 min sessions of the intervention, three times per week.	Current involvement in any other dementia intervention research study.
Be on a stable on medication regimen for at least 4 weeks, prior to study entry.	Unable to understand conversational English, are non-literate or have physical or mental illness severe enough to preclude participation in the study.
	Living in residential care.
**Inclusion criteria—companion**	**Exclusion criteria—companion**
Provide care or support for a person with Parkinson's-related dementia.	Lack capacity to consent to the study to ensure and able to undertake the training and delivery of the intervention.
Be willing and well enough to deliver 30 min sessions of the intervention, three times per week.	Unable to understand conversational English, are non-literate or have physical or mental illness severe enough to preclude participation in the study.
Be familiar with the participant's physical and mental health and able to provide feedback about this to the study team.	A diagnosis of dementia; the presence of dementia in the companion will be determined by self-report as well as clinical impression informed by performance on the MoCA.^21^

DLB, dementia with Lewy bodies; MoCA, Montreal Cognitive Assessment; MCI-PD, Parkinson's disease with mild cognitive impairment; PDD, Parkinson's disease dementia.

We will recruit from centres based at Manchester, Warrington, North East London, and Derby, in the UK. Centres will have established memory or movement disorder clinics and high volumes of people with PDD, MCI-PD or DLB. As recruitment may be influenced by clinic closures during certain times of the year (eg, Christmas and the summer months), recruitment will be carried out over 15 months to obtain an accurate estimate of recruitment rate. The study will be advertised on UK-based charity websites (eg, Parkinson's UK, the Lewy Body Society) and through Join Dementia Research (https://www.joindementiaresearch.nihr.ac.uk). User-friendly information brochures (available as on online supplementary file) will be available at memory and movement disorder clinics and support groups, and information talks will be given at Parkinson's UK community groups. In a recent trial of iCST involving people with non-PD dementias and their companions, the recruitment target (356 dyads over 15 months) was achieved^5^; however, the level of companion burden experienced in the non-PD dementia population may be less compared with that experienced by companions of people with PDD, MCI-PD and DLB, and this may influence recruitment.^14^ Furthermore, Parkinson's-related dementia is much less common compared with dementia of the Alzheimer type.^15^ Therefore, one of the goals of this study is to obtain an accurate estimate of the recruitment rate for participants and their companions.

### Sample size

A predetermined sample size for the feasibility aspects of this study is not required, since inferential statistics will only be exploratory. The sample size is determined by that required to detect a clinical meaningful difference in our primary outcome measure, cognitive function. To determine sample size, we referred to the previous literature^7 8 16^ and, taking a conservative approach, have estimated the standardised effect size on cognition of the parent form of the intervention to be 0.4. Thus, assuming 80% power and a correlation coefficient of 0.5 between baseline and endpoint on cognitive outcomes, the required sample size is 27 completers per group. By enrolling 32 dyads per group, it will allow for a 15% attrition rate (consistent with pilot sample guidance^17^). For the secondary, exploratory outcomes, the proposed sample size of 27 per group is within the recommended guidelines (24–50 participants^18 19^) required to estimate the standard deviation (SD) for a sample size calculation.

### Randomisation and blinding

Eligible dyads will be identified at recruiting centres by trial collaborators (principal investigators) and research nurses and therapists. Participant and companion information sheets outlining the study objectives, risks and benefits will be shared with each dyad. Informed consent from the participant and companion, based on the Good Clinical Practices guidelines published by International Council for Harmonisation,^20^ will be gained during the home screening visit (participant and companion information sheets and informed consent forms are available as on online supplementary file). In line with the aforementioned iCST/CST trials, guidance from the British Psychological Society on evaluation of capacity will be followed. In the event of a participant with PDD/DLB not having capacity at study entry or losing capacity after study entry, the ethics committee has approved the INVEST trial implementer to gain consent from an acceptable, nominated representative. During the home screening, the unblinded trial implementer will administer the Montreal Cognitive Assessment (MoCA)^21^ and Schwab and England Activities of Daily Living Scale^22^ to confirm eligibility and collect demographic data. Once the participant and companion are consented, blinded assessors will conduct baseline assessments in the dyad's home.

Following baseline assessment, the unblinded trial implementer will randomise the dyad to one of two arms: CST-PD or TAU. A tamper proof process of single-strata, blocked randomisation will be applied and communicated via telephone and confirmatory email by an independent arbiter, the Manchester Academic Health Science Centre Clinical Trials Unit. Due to the nature of the intervention, dyads will not be blind to treatment allocation.

Immediately following the intervention period (see below for details), the blinded assessors will visit dyads in their home to conduct post-test assessments. The unblind trial implementer will remind all dyads to maintain the blind during the assessment. Following assessment, blinded assessors will be asked to record the arm to which they believed the dyad was allocated to.

### Active intervention

The active intervention, CST-PD, is based on conventional CST^7^ and iCST^5^ and was developed by means of an iterative process involving significant user, companion and professional input. This adaptation process is described in detail elsewhere and has resulted in a condition-specific cognitive stimulation therapy manual with training guidance. The manual comprises over 60 topics categorised into nine different themes (table 3). Each topic contains several cognitively stimulating activities, for example, discussion suggestions, word association and creative tasks. Activities vary in complexity and can be matched and adapted to suit the needs of the participant. It is recommended that the companion-guided therapy is delivered in 30 min sessions, three times a week over 10 weeks.

**Table 3:** Themes and sample topics from the CST-PD manual

Session theme	Sample topics
Personal life	Childhood, family, relationships
Food	World cuisine, staying healthy, ingredients
Hobbies and leisure	My perfect day, pets, gardening
Art	History of art, Lowry and Turner, architecture
Media and entertainment	Musical instruments, current affairs, technology
Nature	Weather, water, animal kingdom
Seasons	Spring, Summer, Autumn, Winter
Travel and culture	Continents, flags, Blackpool
Games and tasks	Board game, proverbs, colouring and doodling

A specially trained trial implementer (eg, nurse, therapist or researcher) will visit the dyad at home and provide therapy training to the companion. During the training session, the companion will receive the therapy manual, an activity resources pack, the therapy log (companion's diary), information related to the history and objectives of the therapy, and guidance on the nine core principles of the therapy. The trial implementer will guide the participant through a sample session, and the companion will be encouraged to continue with a short session afterwards. The trial implementer will provide constructive feedback to the companion after the session. Once the 10-week intervention period has started, the trial implementer will contact the companion weekly to confirm ongoing consent and adherence and to provide therapy support (if needed). In exceptional circumstances and where telephone assistance is not sufficient (eg, companion doubting confidence in their ability to continue delivering the training), further home visits may be made for ‘refresher’ training sessions. If the dyad is unable to complete any therapy for more than three successive weeks, they will be withdrawn from the trial. To standardise training, the trial implementer will follow a detailed training protocol.

### ‘Treatment as usual’ (control) group

For the purposes of the feasibility evaluation, the inclusion of a control group (ie, TAU) is important because it allows for a more realistic evaluation of the operational aspects (ie, recruitment, retention and randomisation) as well as the implementation of the intervention.^23^ If conducted in an unblinded, non-randomised manner, the outcomes, particularly for a complex psychosocial intervention such as is being tested here, may be quite different. Furthermore, since previous evidence enabled us to power the primary outcome measure, cognitive function, for an evaluation of efficacy, a pilot trial was considered possible and this required inclusion of a control group to account for the natural progression of symptoms during the trial. Specifically, in the TAU comparator arm, dyads will not receive any additional intervention to their standard NHS treatment for PD and cognitive impairment or DLB. Typically, this involves dopamine replacement therapy for the symptomatic relief of Parkinsonism, cognitive enhancing medication (eg, cholinesterase inhibitors) and support from a Parkinson's disease nurse specialist. Since people with Parkinson's-related dementia tend to have significant symptoms in multiple domains, the support of occupational therapist, speech and language therapists and physiotherapists is often required. These interventions will be available to both the TAU as well as those dyads randomised to the active treatment arm. Treatment fidelity will be actively monitored in the control group, as it is in the experimental group, to ensure that the control group do not receive an active intervention component which could reduce the effect size between the experimental and control groups or, similarly, to ensure that an iatrogenic component is not added to the control group which could artificially inflate the difference in outcomes between the two groups.^24^

### Patient and public involvement

Service users, service providers and PPI representatives have been involved in the developmental stages leading up to this study. To ensure that PPI continues to be integrated throughout the INVEST trial, two couples (both involving a person living with PD and their life partner) are active members of the Trial Steering Group (TSG). Additional PPI representatives will be involved in the piloting of interview schedules, advising on the language used to raise awareness of the study, promoting the study through networks and disseminating the research.

### Outcome measures

#### Feasibility and acceptability measures

We shall describe individual demographic and clinical characteristics of participants and companions. We will evaluate several feasibility and acceptability components with a series of outcome measures outlined in table 4. The recruitment and attrition rates to inform subsequent trials will be estimated.

**Table 4:** Description of feasibility measures

Feasibility component	Measure	Described as
Recruitment	Recruitment rate	The number of dyads consented/number eligible
Attrition	Attrition rate	The number of dyads withdrawn/number consented
Therapy adherence	Therapy log (companion's diary)	The total dose (frequency and duration) of the intervention delivered, recorded by the companion after each session
Acceptability of therapy	Therapy log (companion's diary)	Companions will rate the extent to which (in each session) the participant was interested, motivated, gained a sense of achievement; took the initiative and displayed emotional responses. Ratings will be made on a 5-point Likert scale on which ‘1’= strongly disagree and ‘5’=strongly agree.
Acceptability of assessments	Interview record form/focus group	Mixed methods. Quantitative data will include response rates, time taken and level of missing data. Qualitative investigation (assessors interview record form/focus group with blinded assessor) will explore the process: how were the assessments administered, queries raised by respondents, respondents’ markings on the instrument, practical difficulties observed and strategies employed to overcome challenges.

To triangulate the data, qualitative semistructured interviews will be conducted with a small sample following completion of the intervention. The interviews will seek to ascertain acceptability information relating to expectations and experiences of the therapy and barriers and facilitators to completing the therapy. Data will be collected from a purposive sample of 12 dyads, since new themes are reported to occur infrequently beyond this sample size.^25^ The purposive subsample will aim to maximise diversity in dyads’ characteristics to increase the validity and transferability of research findings to other settings. The sample will include (1) people taking part in the therapy, (2) men and women, (3) people of ages crossing the age range of the main sample and (4) people with different levels of cognitive ability (eg, PDD and MCI-PD). Approximately three to four dyads will be invited for interview from each centre.

All audio-recorded interview data will be transcribed verbatim and analysed using thematic analysis or other appropriate analytical processes.

#### Primary and exploratory outcome measures

The piloted efficacy and exploratory outcome measures for the participant are outlined in table 5a and the exploratory outcome measures for the companion in table 5b. The pilot efficacy measure on which the study has been powered is cognitive function as assessed using the Addenbrooke's Cognitive Examination III^26^ and the Everyday Cognition Questionnaire.^27^ Although the pilot trial has been powered on a previously reported effect size, the adaptation of the therapy and single outcome measure means that any potential treatment effects will be interpreted cautiously in order to not mislead the subsequent sample size and power calculation of the full RCT with multiple outcomes.^28^ With this caveat, we will test the hypothesis that compared with the TAU group, the difference in the total cognitive score between baseline and endpoint will be significantly greater in the experimental group. We will undertake a complete case analysis, only including trial completers without missing data, as well as an additional analysis based on last observation carried forward for dyads with missing data. Group differences in the change of outcome measures will be assessed using t-test or analysis of covariance adjusting for baseline differences, including cognitive stage at baseline.

**Table 5:** Primary and exploratory outcome measures

Measure/author	Purpose	Time point
**a) Efficacy and exploratory outcome measures for the participant**		
The Addenbrooke's Cognitive Examination III^26^	Assesses cognitive function	Baseline/post-test
Everyday Cognition Questionnaire^27^	Measures everyday cognition in people with movement disorders	Baseline/post-test
Pill Questionnaire^37^	Measures functional capacity	Baseline/post-test
Lille Apathy Rating Scale^38^	A scale to detect and quantify apathy	Baseline/post-test
Relationship Satisfaction Scale^39^	Assesses level of satisfaction in a relationship	Baseline/post-test
The Brief Resilience Scale^40^	Assesses the ability to recover from stress	Baseline/post-test
Interpersonal Reactivity Index^41^	Measures empathetic concern	Baseline/post-test
Hospital Anxiety and Depression Scale^42^	Assesses depression and anxiety	Baseline/post-test
Parkinson's Disease Questionnaire-39^43^	A Parkinson's disease-specific quality of life measure	Baseline/post-test
Dementia Cognitive Fluctuation Scale^44^	Assesses proxy-reported fluctuations in cognition	Baseline/post-test
The Neuropsychiatric Inventory^45^	Assesses presence or absence of behavioural disturbances	Baseline/post-test
EuroQoL 5D^31^	Used for calculation of quality-adjusted life-years	Baseline/post-test
Client Services Receipt Inventory^30^	Collects information about medications and services used before entering the trial and during the trial	Baseline/post-test
**b) Exploratory outcome measures for the companion**		
Short Form-12 Health Survey^46^	Measures an individual's perception of their physical and mental health	Baseline/post-test
Relationship Satisfaction Scale^39^	Assesses level of satisfaction in a relationship	Baseline/post-test
Brief Resilience Scale^40^	Assesses the ability to recover from stress	Baseline/post-test
Hospital Anxiety and Depression Scale^42^	Assesses depression and anxiety	Baseline/post-test
Zarit Burden Interview^47^	Measures companion burden due to caregiving	Baseline/post-test
Dyadic Relationship Scale^48^	Measures positive and negative aspects of the dyadic relationship	Baseline/post-test
Relatives’ Stress Scale^49^	Measures perceived stress associated with caregiving	Baseline/post-test
Family caregiving role^50^	Measures aspects of caregiving related to satisfaction, love and anger	Baseline/post-test

Ascertaining efficacy of a complex intervention can be challenging since anticipating which outcome measures might be affected by the intervention as either a final or intermediate outcome (eg, mediators or moderators)^29^ can be difficult. Thus, we have carefully considered the underlying theoretical frameworks driving the process and consequently have incorporated several exploratory outcome variables to assist in determining what a clinically meaningful difference in the outcome might be (table 4). Data on exploratory outcome measures will be presented and summarised using means and SDs or alternatives in the case of non-normally distributed data. Data will be compared with previous trials of CST and iCST to understand the possible impact of the adapted therapy. This information can then be used to inform a subsequent full-scale hypothesis testing study designed to detect the smallest clinically meaningful difference.^23^

### Cost-effective analysis

To obtain data on the potential health economic and cost-effectiveness implications of the intervention in a subsequent fully powered RCT, we will assess the use of the Client Services Receipt Inventory^30^ and the EuroQoL 5D^31^ by using baseline and follow-up data to estimate the validity and responsiveness of these questionnaires.

### Adverse events

CST is not known to be associated with adverse events; however, we will monitor for such events. In the unlikely event that an adverse event occurs, the dyad will be directed to relevant health services, and the event will be recorded on a structured form. Advice on how to proceed (ie, to withdraw the dyad temporarily or permanently) with be sought from the chief investigator and sponsor.

## Ethics and dissemination

The trial has received favourable opinion from the research ethics’ committee (15/YH/0351). Protocol amendments will be prepared by the core research team as directed by the chief investigator and TSG. Ethical approval will be sought. The research governance offices and, where applicable, the trial registry, funding body and dyads will be informed of the trial amendments.

### Research governance

The trial is sponsored by the Greater Manchester Mental Health NHS Foundation Trust, UK. The TSG will provide operational management of the trial and supervision on behalf of the trial sponsor and the trial funder (National Institute for Health Research under the Research for Patient Benefit programme) to ensure that the trial is conducted as set out in the MRC's Guidelines for Good Clinical Practice. In this early phase trial, a formal data monitoring committee will not be convened; this function will be provided by the TSG. In relation to data monitoring, the TSG will safeguard the interests of trial dyads, assess the safety and efficacy of the intervention and make recommendations to stop the trial if warranted. The TSG will meet three times per year; terms of reference are available on request. There are no plans for interim analyses at this time.

### Data management

Participant and companion identifiable data will be securely stored in locked cabinet at the University of Manchester. The data will be accessible by the chief investigator (IL), research coordinator (SM) and research assistant (SV). Coded research data (blind to which arm is the intervention arm) will be made available to the independent statistician (BF) to conduct the primary analysis. The code will be broken only after the primary analysis has been completed.

### Dissemination policy

Findings will be submitted to high-impact peer-reviewed journals, presented at national international academic conferences and communicated to other universities. Publication authorship will be based on the International Committee of Medical Journal Editors’ criteria. Ongoing dissemination to clinicians and academics will be facilitated through the development and regular maintenance of a project-specific website. A lay summary will be sent to dyads (if requested) and presented at community engagement events.

### Summary

This mixed-methods protocol describes an early phase trial designed to primarily evaluate the feasibility and acceptability of CST-PD and whether there is sufficient evidence of clinical efficacy to justify a subsequent larger RCT. The protocol is congruent with the UK's MRC's updated guidance on ‘Developing and Evaluating Complex Interventions’^10^ and serves to enhance the design of a subsequent definitive large-scale trial.

### Trial status

The INVEST study and trial started on 18 January 2016 and will be recruiting dyads from 1 March 2016 to 30 June 2017. The end date for data collection is 30 October 2017.

10.1136/bmjopen-2017-016801.supp2 Supplementary material 2

10.1136/bmjopen-2017-016801.supp3 Supplementary material 3

10.1136/bmjopen-2017-016801.supp4 Supplementary material 4

10.1136/bmjopen-2017-016801.supp5 Supplementary material 5

10.1136/bmjopen-2017-016801.supp6 Supplementary material 6

## Supplementary Material

Reviewer comments
