# Supplementary material for: Psychosocial therapy for Parkinson's-related dementia: study protocol for the INVEST randomised controlled trial
Source: BMJ Open. 2017 Jun 19;7(6):e016801. doi: 10.1136/bmjopen-2017-016801 (PMC5726123; doi:10.1136/bmjopen-2017-016801)
Supplement: Supplementary material 1 [file bmjopen-2017-016801supp001.pdf]

We welcome your help

Assessing iCST for people with movement disorders and their caregivers is extremely important to us. For example, we would like to know if the adapted therapy is acceptable and if it meets peoples' needs.

The INVEST project aims to gather this information by conducting an NIHR Research for Patient Benefit Programme funded study. If you would be interested in taking part and using the therapy at home please contact:

Dr Sheree McCormick: Tel: 0161 306 7494

sheree.mccormick@manchester.ac.uk

Ms Sabina Vatter: Tel: 0161 306 7913

sabina.vatter@manchester.ac.uk

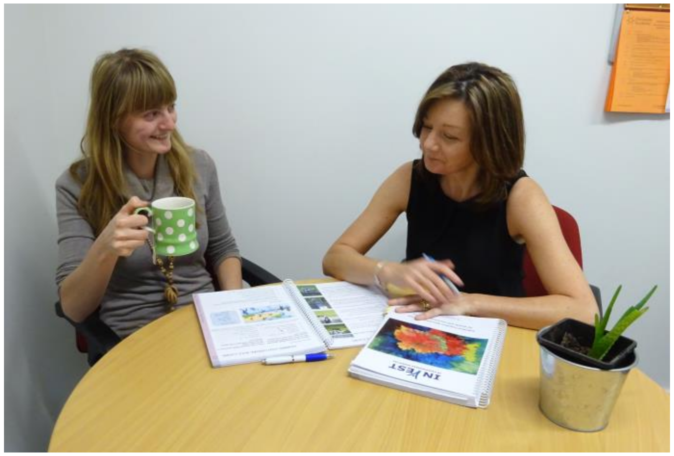

If the adapted iCST is successful, it will improve the quality of life for people with movement disorders and their caregivers.

INVEST
IN PARKINSONIAN DEMENTIAS

Individual Cognitive Stimulation Therapy (iCST) for People with Movement Disorders

This information booklet is provided for:

- Individuals with Parkinson's disease dementia or Dementia with Lewy bodies
- Family members
- Caregivers

.

MANCHESTER 1824
The University of Manchester

NHS
National Institute for Health Research

The Challenge

People with Parkinson's disease are 20 – 40% more likely to develop dementia compared to people without the disease. Dementia, or mild cognitive impairment, in Parkinson's disease can cause mental impairment, such as problems with memory and attention, and can have a negative impact on people with the disease and their caregivers.

Cognitive Stimulation Therapy has been demonstrated to help overcome some of the mental challenges that can decrease quality of life for people with dementia and their caregivers. The therapy involves engaging in structured, enjoyable activities that support memory and stimulate communication and everyday planning.

Cognitive Stimulation Therapy is the only non-drug therapy widely used in the NHS. However, it is unknown whether this therapy is suitable for people with Parkinson's disease dementia or Dementia with Lewy bodies.

Cognitive Stimulation Therapy can help overcome some of the mental challenges associated with dementia and improve quality of life

A Solution

The INVEST study has developed Individual Cognitive Stimulation therapy (iCST) for people with Parkinson's disease dementia or Dementia with Lewy bodies. iCST is delivered at home by the caregiver. The therapy involves structured activities that are interesting and enjoyable. The manual contains guides for each task and no additional equipment is required.

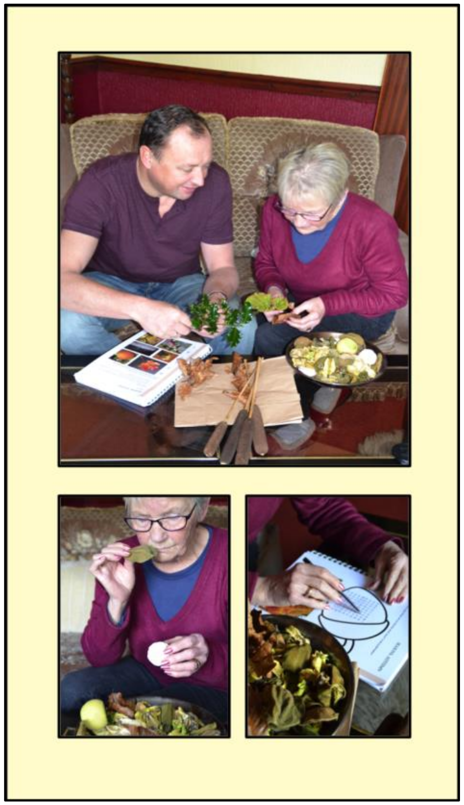

A typical therapy session might involve:

Selecting a topic of your choice and sharing your opinions about the photographs and images provided.

Recalling happy memories linked to the topic.

Using different senses to think about the topic.

If desired, completing more challenging activities related to the topic.
