# Supplementary material for: Psychosocial therapy for Parkinson's-related dementia: study protocol for the INVEST randomised controlled trial
Source: BMJ Open. 2017 Jun 19;7(6):e016801. doi: 10.1136/bmjopen-2017-016801 (PMC5726123; doi:10.1136/bmjopen-2017-016801)
Supplement: Supplementary material 3 [file bmjopen-2017-016801supp003.docx]

**INFORMATION SHEET FOR CAREGIVERS**

**Psychosocial Therapy to Benefit Patients with Parkinson’s-related Dementia: Development of an individualised Cognitive Stimulation Therapy programme.**

**Invitation to participate in a research study**

You are being invited to take part in a research study. Before you decide, it is important for you to understand why the research is being done and what it will involve. Please take time to read the following information carefully and discuss it with others if you wish. Ask us if there is anything that is not clear or if you would like more information. Take time to decide whether or not you wish to take part. Thank you for reading this information sheet.

**What is the purpose of the study?**

In recent years, Cognitive Stimulation Therapy (CST) groups have shown to be an enjoyable and beneficial therapy for people with memory problems. This project will show whether individualised (one-to-one) CST can be tailored for people who have problems with memory and also have difficulties with movement (for example, a tremor or muscle stiffness).

**Why have I been chosen?**

You have been invited to take part because of your support for a person who has had a diagnosis of a movement disorder with memory problems. We need up to seventy-six people in total who are caregivers for people with movement disorders and memory problems.

**What happens in individualised cognitive stimulation therapy (iCST)?**

iCST sessions will last for 30 minutes and take place three times a week for ten weeks. The activities will include, for example, discussion of art and current affairs. The idea is to keep the mind active through enjoyable activities. We will ask you to guide your relative/friend through the activities and will provide you with the training and support you need to be able to do so. We will ask you to complete provide some about each of the activities.

**Do I have to take part?**

It is up to you to decide whether or not to take part. If you do decide to take part you will be given this information sheet to keep and be asked to sign a consent form. If you decide to take part you are still free to withdraw at any time without giving a reason. A decision to withdraw at any time, or a decision not to take part, will not affect the standard of care your relative / friend receives.

**What happens to me if I take part in this study?**

This study is a randomised trial. We need to see whether iCST is better than no treatment, so we need to compare any changes experienced by people receiving iCST to those receiving no additional therapy. The fairest way of doing this is to select people for the group by chance; everyone agreeing to take part will have a 50:50 chance of receiving iCST. The decision is made by an independent computer, which will not have any identifying information about you or your relative/friend.

If you decide to take part, stage one of the study will last for a time period of about fourteen weeks. Following discussion of any questions you may have with a researcher, and signing the consent form, all participants will be allocated to the iCST group or the group that doesn’t receive iCST, then:

1. If you are in the iCST group you will be asked to meet with a researcher for a half day of training to lead the iCST sessions. The training can take place in your home or at the University of Manchester, if you prefer. This will happen around two weeks before starting the therapy, to give you time to think, read and ask any questions you might have.
2. All participants will be asked to meet with a researcher for between two / two-and-a-half hours to complete an interview and some questionnaires covering your quality of life, mood, and about the relationship you have with your relative/friend. You will also be asked to complete some health questions about your relative/friend. The time stated to complete the interviews and assessments is an estimate; you and your friend/relative may take as many breaks as you want or feel necessary, and even complete the process over two sessions if preferred.
3. *For the iCST group:* complete three sessions of iCST, each lasting 30 minutes, per week, for ten weeks, *or for the group that doesn’t receive iCST:* continue your regular daily activities with your relative/friend without the therapy sessions.
4. All participants will be asked to repeat the questionnaires and assessments with the researcher after 10 weeks. This is to see whether any of these factors changed for people who received the iCST intervention. At this point (if your relative/friend received iCST) you may also be asked to complete a separate interview with the researcher to record what you thought about the therapy. It is important for you to know that this interview will be voice recorded for analysis at a later date.

Usually, the researcher will come to your home or the home of your relative/friend, but will be happy to meet you elsewhere if you would prefer. The researcher will meet with and interview your relative/friend at the same time as you are completing the questionnaires.

**Optional parts of the study:**

1. After completing the study, participants allocated to the control group will be offered an opportunity to experience the therapy. A researcher will visit the participants at home and provide therapy training. Following therapy training the participants will have access to on-going support from the research team for a period of 10 weeks. No data will be collected during or after this time, the therapy is offered as a courtesy to the control group.
2. At the end of the study, we may ask some participants to take part in focus groups. These will last around half a day and will be led by the research team. The purpose of the focus groups is to ask for your feedback on how the therapy might be improved or adapted. Refreshments will be provided for everyone during breaks. It is important for you to know that the focus group will be voice recorded for analysis at a later date.

**Expenses**

Any reasonable travel expenses you incur will be reimbursed.

**What do I have to do?**

Taking part in the study does not involve any lifestyle restrictions or changes. You can carry on with your everyday activities as normal. All we ask is that you keep your appointments with us on the days of the assessments, interviews and the focus group.

**What are the possible disadvantages and risks of taking part?**

iCST aims to be stimulating and enjoyable. Sessions involve discussing themes such as food, childhood and current affairs and the level of risk in taking part is therefore minimal. You will be given guidance on what to do if your relative/friend becomes anxious or distressed during sessions. If delivering the intervention really does not suit you or the intervention does not suit your friend/relative, you are free to finish or withdraw at any point.

**What are the possible benefits of taking part?**

If you decide to take part we hope that it might be enjoyable for you. There may be no direct benefit to participating but we hope that the stimulating activities might improve your quality of life. The information that we get from this study may help us to develop a programme that, in the future, could help to treat people with movement disorders and memory problems. As such, your contribution would be valuable.

**Will my taking part in the study be kept confidential?**

All information which is collected about you during the course of the study will be kept strictly confidential. All data is stored without any identifying details under secure conditions.

**What will happen if I don’t want to carry on with the study?**

You will be free to withdraw from the study at any time, without giving a reason. Withdrawing from the study will not affect the standard of care you receive. We will need to use any data collected in the study, up to the point of withdrawal.

**What if something goes wrong?**

If you are harmed by taking part in this study, there are no special compensation arrangements. If you are harmed due to someone’s negligence, then you may have grounds for a legal action, but you may have to pay for your legal costs.

Regardless of this, if you wish to make a complaint about any aspect of the way you have been approached or treated during the course of this study, the normal National Health Service complaints procedures should be available to you. If you are unhappy or dissatisfied about any aspect of your participation, we would ask you to tell us about this in the first instance, so that we can try to resolve any concerns and find a solution.

**Who is organising and funding the research?**

The research is funded by the National Institute for Health Research ‘Research for Patient Benefit’ scheme. This funding covers the running costs of the research project and is led by Dr. Iracema Leroi, who is an Old Age Consultant Psychiatrist for Manchester Mental Health and Social Care NHS Trust and a Senior Lecturer in Psychiatry at the University of Manchester. The study is in collaboration with researchers from University College London.

**Consent form for use of audio recording and direct quotes:**

An audio recording of the interviews and focus groups will be taken. The purpose of the audio recording is to help the researchers to identify the most important aspects of the discussion at a later date. At any point, you may request that the audio recording is paused, or temporarily stopped. However, we will require use of the recording to this point. We may use direct quotes from the audio recording in our publications but we will do so in an anonymised form so that your identity is protected.

**What will happen to the results of the research?**

The results from this study will help us decide whether or not to run a larger study to help people with movement disorders and cognitive impairment. The results will be published by the Department of Health, and in relevant health journals. No participants will be identified in any publication arising from the study, without their written consent. We will make arrangements for participants to be informed of the progress of the research and the results through newsletters and local meetings. If you would like a short summary at the end of the study please let the research team know. Participants in the control arm will also be able to access the research findings and receive a short summary of the results if they wish. Upon completion, a summary can be posted at your home address.

**Who has reviewed the study?**

All NHS research is looked at by an independent group of people, called a Research Ethics Committee to protect your safety, rights, well-being and dignity. This study has been reviewed and been given a favourable opinion by the NRES Yorkshire & The Humber-Bradford Leeds Ethics Committee.

**Who can I contact for further information?**

If you would like further information or have any questions about the study please contact one of the study researchers listed below:

Dr Sheree McCormick

Research Associate

Tel: 0161 306 7494

[sheree.mccormick@manchester.ac.uk](mailto:sheree.mccormick@manchester.ac.uk)

Ms Sabina Vatter

Research Assistant

Tel: 0161 306 7913

[sabina.vatter@manchester.ac.uk](mailto:sabina.vatter@manchester.ac.uk)

**Or if you have any complaints about this study please contact:**

If you have a concern about any aspect of this study you should ask to speak to one of the Research Team: Dr Sheree McCormick/ Sabina Vatter (0161 306 7494 / 0161 306 7913) or the Chief Investigator Iracema Leroi (tel: 0161 306 7944) who will do their best to help.

If there are any issues regarding this research that you would prefer not to discuss with the research team, please contact the Research Practice and Governance Co-ordinator by either writing to ‘The Research Practice and Governance Co-ordinator, Research Office, Christie Building, The University of Manchester, Oxford Road, Manchester, M13 9PL’, by emailing research.complaints@manchester.ac.uk or by telephoning 0161 275 8093.

**The following services are also available for help and advice should you require it:**

**Greater Manchester Mental Health NHS Foundation Trust Patient Advice and Liaison Service (PALS)**

Telephone: 0161 882 2084/2085 Patient Advice and Liaison Service Mobile: 07815 284660 Greater Manchester Mental Health NHS FT, E-mail: PALS@mhsc.nhs.uk 11th Floor, Hexagon Tower Crumpsall Vale Manchester M9 8GQ

**Thank you for considering taking part in this research study.**
