# Supplementary material for: Psychosocial therapy for Parkinson's-related dementia: study protocol for the INVEST randomised controlled trial
Source: BMJ Open. 2017 Jun 19;7(6):e016801. doi: 10.1136/bmjopen-2017-016801 (PMC5726123; doi:10.1136/bmjopen-2017-016801)
Supplement: Supplementary material 4 [file bmjopen-2017-016801supp004.doc]

##### CONFIDENTIAL

**Project Title:**

**Psychosocial Therapy to Benefit Patients with Parkinson’s-related Dementia: Development of an individualised Cognitive Stimulation Therapy programme.**

**Chief Investigator:** Dr. Iracema Leroi

**Participant ID:**

# ***Please initial box***

| 1. | I confirm that I have read and understood the Participant Information Sheet dated 14^th^ of February 2017 (version 1.3) for the above study and have had the opportunity to ask questions. |  |
| --- | --- | --- |
| 2. | I understand that my participation is voluntary and that I am free to withdraw at any time, without giving any reason, without my medical care or legal rights being affected. |  |
| 3.  4.  5.  6.  7.  8.  9. | I agree to take part in the above study.  If allocated to the treatment group, I agree to complete the therapy sessions three times per week for ten weeks.  I agree to take part in the interviews and understand they will be audio recorded.  I agree to the use of anonymised quotes in publications.  I agree to my GP being informed of my participation in the study.  I understand that if my capacity to make my own decisions changes during the study period, members of the research team may seek advice from a Consultee about using data collected from me for the purposes of the study and to ask about my continuation in participating.  a) I agree to be contacted regarding participation in the focus group that will take place near the end of the study.  (Optional) Yes or No: ……………… |  |
|  | b) I agree to the focus group consultation being audio recorded.  c) I understand that if I withdraw during or after the focus group, the audio recording will still be used as it will contain information provided by other people. |  |
| 10. | I agree to be contacted about future studies:  (Optional) Yes or No: ……………… |  |
|  |  |  |

______________________ ____________ ________________________

Name of participant Date Signature

______________________ ____________ ________________________

Name of researcher Date Signature
