# Supplementary material for: Psychosocial therapy for Parkinson's-related dementia: study protocol for the INVEST randomised controlled trial
Source: BMJ Open. 2017 Jun 19;7(6):e016801. doi: 10.1136/bmjopen-2017-016801 (PMC5726123; doi:10.1136/bmjopen-2017-016801)
Supplement: Reviewer comments [file bmjopen-2017-016801.reviewer_comments.pdf]

## PEER REVIEW HISTORY

BMJ Open publishes all reviews undertaken for accepted manuscripts. Reviewers are asked to complete a checklist review form (**http://bmjopen.bmj.com/site/about/resources/checklist.pdf**) and are provided with free text boxes to elaborate on their assessment. These free text comments are reproduced below.

## ARTICLE DETAILS

| | |
|---|---|
| **TITLE (PROVISIONAL)** | Psychosocial therapy for Parkinson's-related dementia: Study protocol for the INVEST randomised controlled trial |
| **AUTHORS** | McCormick, Sheree; McDonald, Kathryn; Vatter, Sabina; Orgeta, Vasiliki; Poliakoff, Ellen; Smith, Sarah; Silverdale, Monty; Fu, Bo; Leroi, Iracema |

This article was not externally reviewed at BMJ Open. Protocols that have been independently assessed prior to submission to BMJ Open are usually fast-tracked to publication on the grounds that further substantial changes will not be possible. This independent assessment will usually be external, independent review for both a substantial grant award from a non-commercial or government funder and ethics approval.
